# Synthesis of multivalent host and guest molecules for the construction of multithreaded diamide pseudorotaxanes

**DOI:** 10.3762/bjoc.8.24

**Published:** 2012-02-09

**Authors:** Nora L Löw, Egor V Dzyuba, Boris Brusilowskij, Lena Kaufmann, Elisa Franzmann, Wolfgang Maison, Emily Brandt, Daniel Aicher, Arno Wiehe, Christoph A Schalley

**Affiliations:** 1 Institut für Chemie und Biochemie der Freien Universität Berlin, Takustr. 3, 14195 Berlin, Germany; 2 Pharmazeutische und Medizinische Chemie, Universität Hamburg, Bundesstr. 45, 20146 Hamburg, Germany; 3 Biolitec research GmbH, Otto-Schott-Str. 15, 07745 Jena, Germany; 4 Charité – Universitätsmedizin, International Graduate Program Medical Neurosciences, Charitéplatz 1, 10117 Berlin, Germany; 5 WITEGA Laboratorien Berlin-Adlershof GmbH, Magnusstr. 11, 12489 Berlin, Germany

**Keywords:** multivalency, pseudorotaxanes, Sonogashira coupling, supramolecular chemistry, tetralactam macrocycles

## Abstract

A series of di-, tri- and tetravalent axles and wheels for the synthesis of pseudorotaxanes bearing the tetralactam macrocycle/diamide axle binding motif was prepared. Starting from iodinated monovalent precursors, Sonogashira cross-coupling reactions were utilized to couple the binding sites to appropriate spacer groups. Through this “Lego” or “toolbox” approach, the convergent synthesis of host and guests with a well-defined number of the binding sites is possible. In addition, the spatial arrangement of the binding sites can be controlled through the quite rigid connections between linker and binding sites. Although a quantitative assessment of binding strengths was not possible by NMR titration experiments, typical and significant shifts of the signals of the diamide moiety indicate qualitatively the formation of pseudorotaxanes from the axle and wheel precursors.

## Introduction

Synthetic supramolecular complexes have the great potential to put those concepts to the test that govern much of the noncovalent chemistry in nature. Among these concepts are not only molecular recognition and the noncovalent bonds themselves, but also self-assembly, self-sorting, templation and multivalent binding [1–8]. Consequently, the reductionist investigation of synthetic supramolecules can help us to understand biological systems better. Such a synthetic approach can also help in the investigation of multivalent binding [9–12], because the number of binding sites can be altered at will, and studies can be done with a suitable series of host and guest molecules in which the nature and number of binding sites is systematically varied.

Interlocked molecules [13–28] are interesting not only because of their particular topology or the mechanical bond, but also as they have been intensely investigated with respect to the construction of molecular machines [29–32]. The mechanical bond appears particularly suited for this goal, because it connects the axle and wheel strongly, but leaves freedom for the relative movement of the two components. Pseudorotaxanes are the precursors for both rotaxane syntheses by stoppering reactions or catenanes by macrocyclization. The use of weak interactions, e.g., metal complexation [33–47], charge-transfer interactions [48–63], or hydrogen bonding [64–79], between the single building blocks is necessary for efficient templating effects, which aim at assembling higher-order molecular architectures. The synthesis of a multiply threaded architecture [80–81] thus requires multivalent wheel and axle components as precursors, which are also interesting with respect to their binding properties. Among the examples of such multivalent pseudorotaxanes [80–82], the “molecular elevators” reported by Stoddart et al. [83–84] are particularly fascinating, because they combine multivalency with the ability of a molecular device to respond to external stimuli, in this case to acids and bases, which induce motion of the wheel and axle components relative to each other.

**Figure 1:**
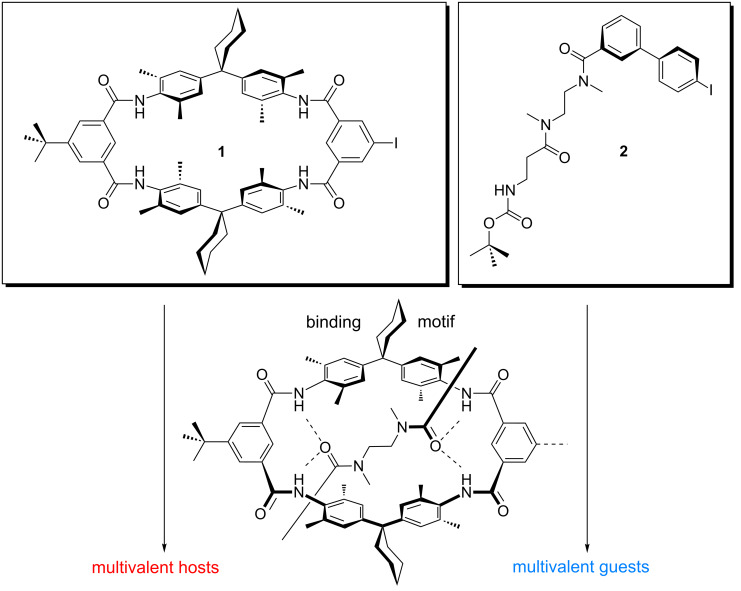
Hunter/Vögtle-type tetralactam macrocycle **1** bearing an iodo substituent at one of the isophthaloyl groups, and the diamide axle piece **2**. The iodoaryl groups in both building blocks are suitable for Sonogashira cross-coupling reactions. Therefore, these monovalent precursors can be connected to different suitable spacers so that a "toolbox" of multivalent hosts and guests can be synthesized convergently. The center shows the binding motif, which connects the axle and wheel by four N–H···O hydrogen bonds.

Tetralactam macrocycles (TLMs) [65–66] have widely been used in the synthesis of amide catenanes and rotaxanes [85–92] and represent excellent hosts for dicarbonyl compounds [93–101]. They bear four converging amide groups, which in each case can form hydrogen bonds to suitable axle molecules in aprotic and not too strongly competitive solvents such as CH_2_Cl_2_ or CHCl_3_. In this contribution, we report the synthesis and binding behaviour of di-, tri- and tetravalent diamide-axle–TLM complexes. The design is based on the two building blocks **1** and **2** (Figure 1). Compound **1** is the wheel component, and **2** is the axle, which comprises a diamide moiety that binds to the wheel as indicated in the center of Figure 1 through the formation of four (wheel)N–H···O=C(axle) hydrogen bonds. Through the iodine substituents, both building blocks can be connected to appropriate spacers in Sonogashira cross-coupling reactions [102].

This coupling strategy creates rather rigid connections to the spacers and helps in reducing the entropic penalties that arise from conformational fixing of the spacers upon multivalent binding. The building blocks were chosen based on simple force-field calculations of the resulting pseudorotaxanes and permit us to synthesize a series of different hosts and guests in a convergent way. Thus, a "toolbox" [103–104] of multivalent host and guest molecules becomes available with this synthetic strategy. In the future, the pseudorotaxanes designed here should be easily converted into rotaxanes after cleavage of the Boc protective group at the axle ends and attachment of stopper groups to the terminal amines.

## Results and Discussion

### Synthesis of monovalent precursors

Although aryl bromides, triflates and sometimes even chlorides react efficiently in Sonogashira cross-coupling reactions, our previous studies [103–104] showed that only the iodinated TLM, and in some rare cases the corresponding triflate-substituted wheel, is reactive enough to provide sufficiently high yields. This is particularly important when the same precursor is to be multiply connected to the same spacer. Low-yielding reaction steps would result in mixtures of the desired compounds with incompletely substituted side products. Therefore, iodo-substituted TLM **1** was prepared according to well-documented literature procedures [64–103] and used for the cross-coupling reactions in this study.

**Scheme 1:**
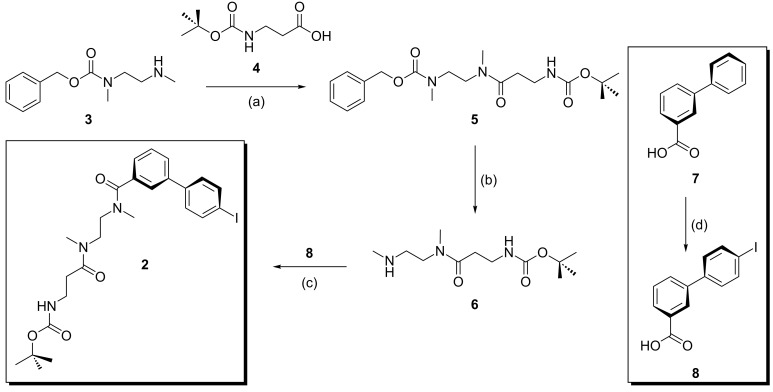
Synthesis of the monovalent diamide axle **2**, which was used for Sonogashira coupling to the appropriate spacers: (a) EDC, HOBt, DMF, 22 h, 92%; (b) H_2_, Pd/C, EtOH, 3 d, 98%; (c) EDC**^.^**HCl, HOBt, DMF, 24 h, 73%; (d) I_2_, PIDA, AcOH/Ac_2_O, 1 h, 67%; (EDC = 1-ethyl-3-(3-dimethylaminopropyl)carbodiimide, HOBt = 1-hydroxybenzotriazole, DMF = *N*,*N*'-dimethylformamide, PIDA = phenyliodine diacetate).

The synthesis of the iodo-substituted monovalent diamide axle centerpiece **2** was realized by the four-step synthesis shown in Scheme 1. The free amino group in mono-Cbz-protected *N*,*N*'-dimethylethylene diamine **3** [105] was elongated with the commercially available *N*-Boc-protected β-alanine **4** in the presence of EDC and HOBt as activating coupling reagents. This step provides the basis for future stopper attachment to the axle termini, as mentioned above. Product **5** was formed with a yield of 92% without the need for time-consuming purification steps. This molecule now contains two orthogonal protecting groups, and hydrogenation of **5** deprotects the amino group at the *N*,*N*'-dimethylethylene diamine site to yield **6** in 98% yield. The iodo-substituted acid **8** can easily be prepared under mild conditions from the iodine-free precursor **7** [106] by using I_2_ and phenyliodine diacetate (PIDA) and is then available for amide coupling with **6** yielding binding site **2** in 73% yield. Since an excess of **7** was used and the 2’/6’-positions are sterically hindered, only monoiodination in the 4’-position was observed. This synthetic pathway thus gives reasonable overall yields.

It should be mentioned that the diamide moiety bears two tertiary amides. All attempts to prepare a similar axle with secondary amide groups failed because of the low solubility of the products. This is an important aspect, because the threading of the station into the TLMs requires noncompetitive solvents such as CH_2_Cl_2_ or CHCl_3_. Consequently, any polar aprotic solvent, such as acetone, acetonitrile, DMF or DMSO, which would solubilize the axles sufficiently well, would interfere strongly with pseudorotaxane formation. The tertiary amides are much more soluble and, therefore, appear to be the more appropriate binding site. However, the better solubility comes at a price. While secondary amides prefer the *trans*-conformation, in which the carbonyl oxygen and the NH proton diverge, the tertiary amides do not exhibit a similarly strong preference for one of the conformations. The axle binding sites thus exist in equilibrium between (*trans*,*trans*)*-*, (*trans*,*cis*)*-* and (*cis*,*cis*)*-*isomers in solution, which complicates the analysis of the binding properties.

### Synthesis of multivalent wheels

**Scheme 2:**
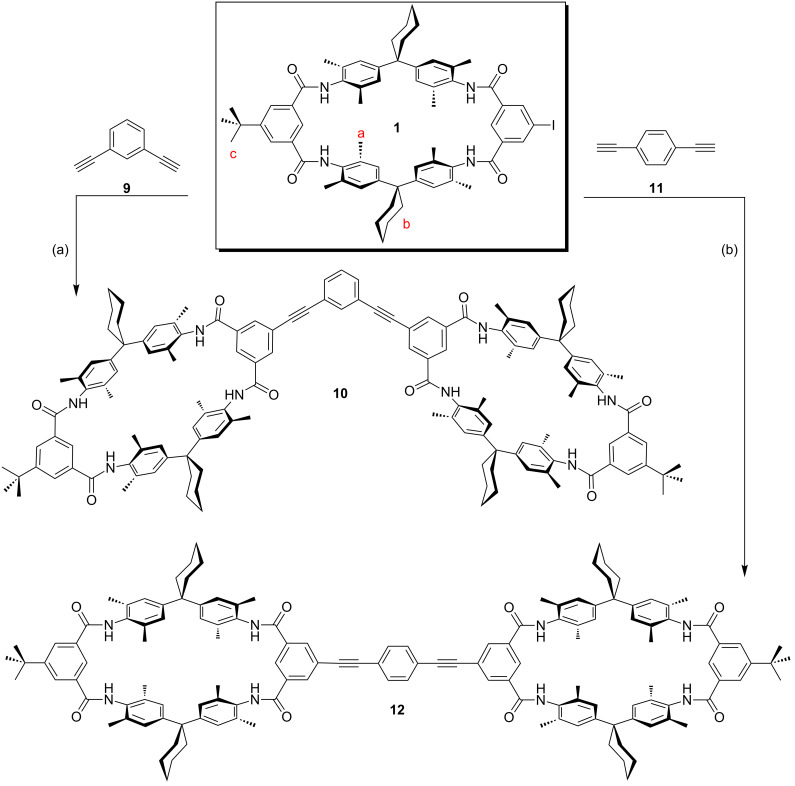
Synthesis of divalent wheels from TLM **1**: (a), (b) (Ph_3_P)_2_PdCl_2_, CuI, PPh_3_, NEt_3_, DMF, 25 °C, 24 h, 90% (**10**), 78% (**12**).

**Scheme 3:**
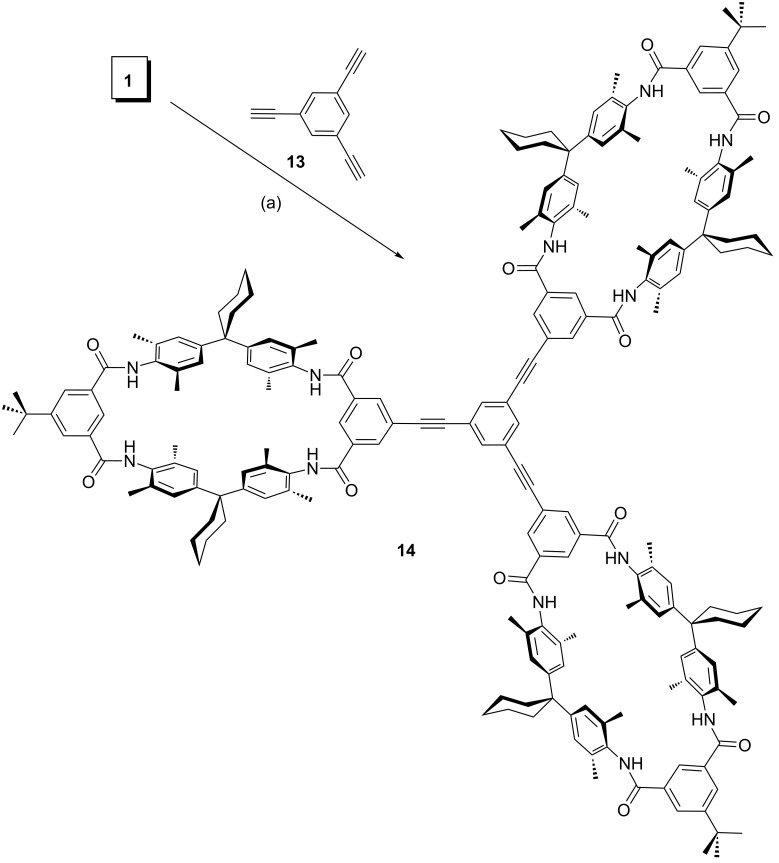
Synthesis of trivalent wheel **14** from TLM **1**: (a) (Ph_3_P)_2_PdCl_2_, CuI, PPh_3_, NEt_3_, DMF, 25 °C, 24 h, 40% (**14**). Compound **14** was described previously [103] and is included to complete the series.

Monovalent axle **2** and TLM **1** are designed to give a good complementary fit, when both are connected to the same flat spacer molecules through Sonogashira cross-coupling reactions. Therefore, ethynyl-substituted benzene spacers **9**, **11** and **13** were used to synthesize a bent divalent (**10**), a linear divalent (**12**), and a trivalent wheel (**14**) [103] (Scheme 2 and Scheme 3). From the enormously broad choice of different Pd-catalysts and reaction conditions, we used (Ph_3_P)_2_PdCl_2_ and CuI in the presence of PPh_3_ as the coligand, NEt_3_ as the base, and DMF as the solvent. This protocol was successfully applied previously for the synthesis of **14** [103]. The yields of 90% and 78% obtained for the divalent hosts **10** and **12**, respectively, were even higher than that for **14** (40%).

**Scheme 4:**
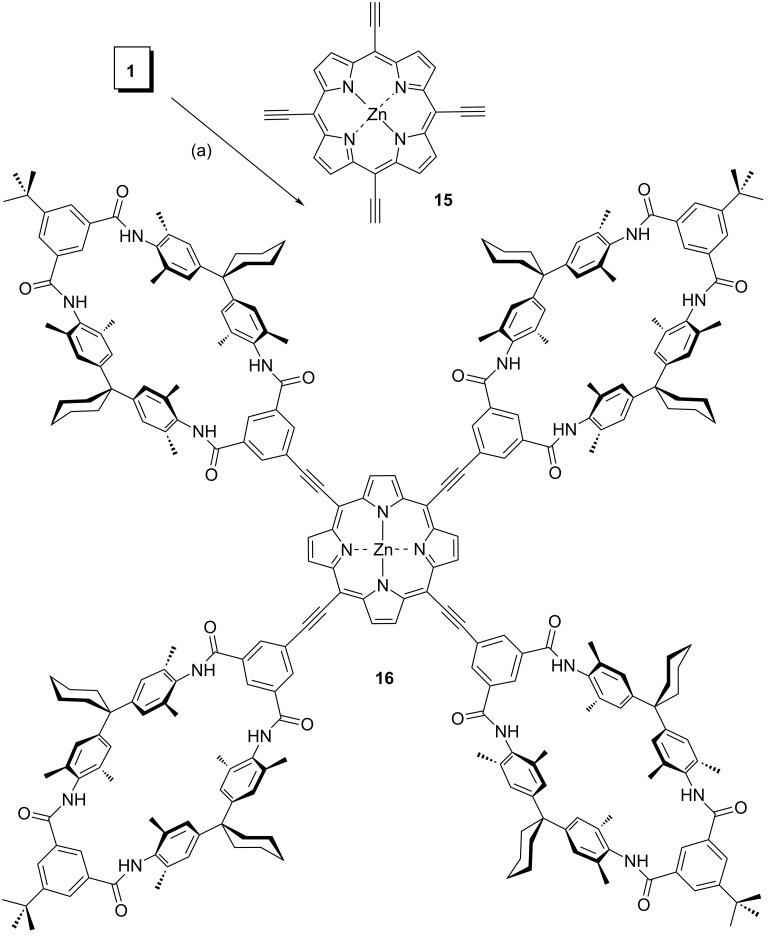
(a) Pd_2_(dba)_3_, AsPh_3_, NEt_3_, DMF, 120 °C, 12 h, 7% (**16**).

All attempts to use the same conditions for the fourfold coupling of **1** to **15** to synthesize tetravalent wheel **16** were unsuccessful, and we finally used another procedure for the cross-coupling reaction [107]. Because the Cu(I) catalyst may interfere with the Zn core of porphyrin **15** or lead to Glaser coupled side-reaction products, a copper-free Sonogashira procedure [107–108] employing Pd_2_(dba)_3_ as the catalyst and AsPh_3_ as the coligand in NEt_3_/DMF was applied (Scheme 4). This reaction unfortunately provided only 7% of the desired tetravalent wheel **16**; however, this amount sufficed for characterization by ^1^H NMR and ESI mass spectrometry. There may be several reasons for this observation. One reason for the low yield may be solubility, which in our experience is always low for tetralactam wheels connected through nicely stacking spacers.

### Synthesis of multivalent axles

**Scheme 5:**
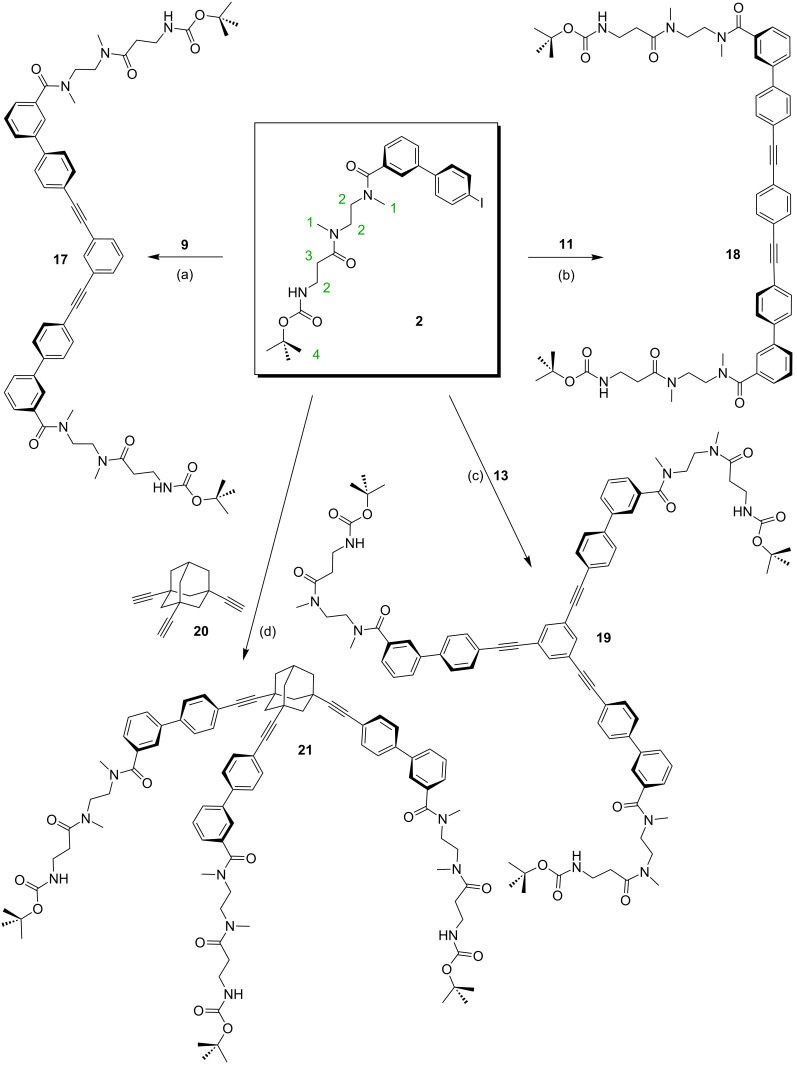
Synthesis of a series of multivalent guests starting from the axle **2**. (a), (b), (c), (d): Pd_2_(dba)_3_, CuI, PPh_3_, NEt_3_, DMF, 70 °C, 3 d; 32% (**17**), 37% (**18**), 24% (**19**), 64% (**21**), respectively.

In order to prepare the multivalent axles that fit to the wheels described above, the same spacers **9**, **11** and **13** were also used for the synthesis of multivalent guests **17**–**19**, respectively (Scheme 5). In addition, a triethynyl-adamantane derivative **20** [109–110] was employed in order to prepare an axle component with a slightly different, nonflat spacer geometry.

**Scheme 6:**
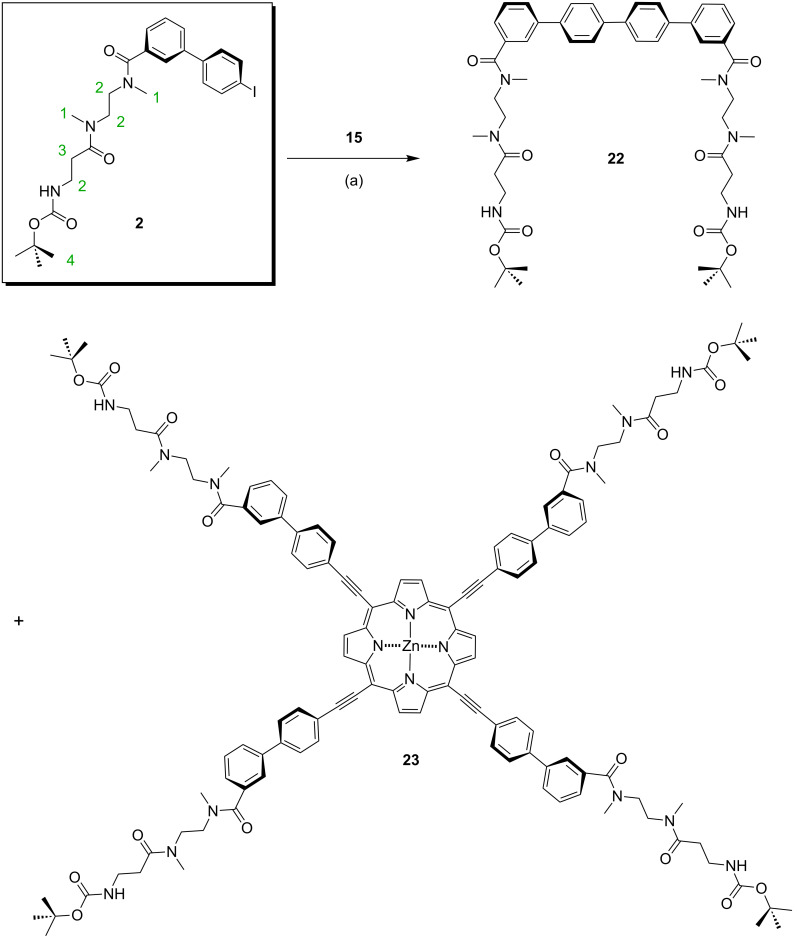
Synthesis of the tetravalent axle **23** and its divalent side product: (a) Pd_2_(dba)_3_, AsPh_3_, NEt_3_, DMF, 80 °C, 2 d, 37% (**22**), 17% (**23**).

For the axle preparation, neither the use of (Ph_3_P)_2_PdCl_2_/CuI/PPh_3_ nor that of Pd_2_(dba)_3_/AsPh_3_ provided the desired products, and therefore the conditions of the Sonogashira cross-coupling reactions had to be optimized again. Finally, the mixture of Pd_2_(dba)_3_/CuI/PPh_3_ gave the di- and tritopic axles **17**–**19** and **21** in DMF/NEt_3_ with decent yields when the temperature was raised to 70 °C. The copper-free Sonogashira procedure was again applied for the synthesis of tetravalent guest **23** (Scheme 6) and yielded 17% of the product. Interestingly, a homocoupling of axle **2** gave rise to divalent axle **22**, which was isolated as a side product in 37% yield. This molecule may be useful for other divalent hosts, such as two macrocycles connected through a butadiyne spacer or a thiophene unit. Such hosts have been reported previously [103] and are not included here.

### Formation of pseudorotaxanes

In order to determine the binding strengths quantitatively, NMR titrations, dilution experiments, and ITC experiments were attempted. The NMR titrations suffer from the fact that different conformations of the tertiary amide groups hamper an in-depth evaluation of the titration data. The diamide *N*-CH_3_ and *N*-CH_2_ protons appear with four sets of signals, one for the (*trans*,*trans*)-, two for the asymmetric (*trans*,*cis*)- and one for the (*cis*,*cis*)-isomer. This, and significant signal overlaps, make it impossible to quantify the NMR titration data. For ITC measurements, the concentration range in which one can expect reasonable heats to evolve upon binding could not be reached, due to the low solubility of the wheel components. Furthermore, mass spectrometry was attempted in order to show the formation of 1:1 complexes qualitatively, but this was without success. This does not come as a surprise in view of previous theoretical calculations on amide/tetralactam macrocycle complexes [111], which show that simple amide axles dethread in the gas phase because of a favourable entropy term arising from the increase in particle number upon complex dissociation. This entropic contribution overcompensates for the enthalpic contribution to the binding. As the calculations were done for monovalent complexes and monoamide, we nevertheless attempted to ionize complexes of our di- and trivalent systems, but unfortunately without success.

**Figure 2:**
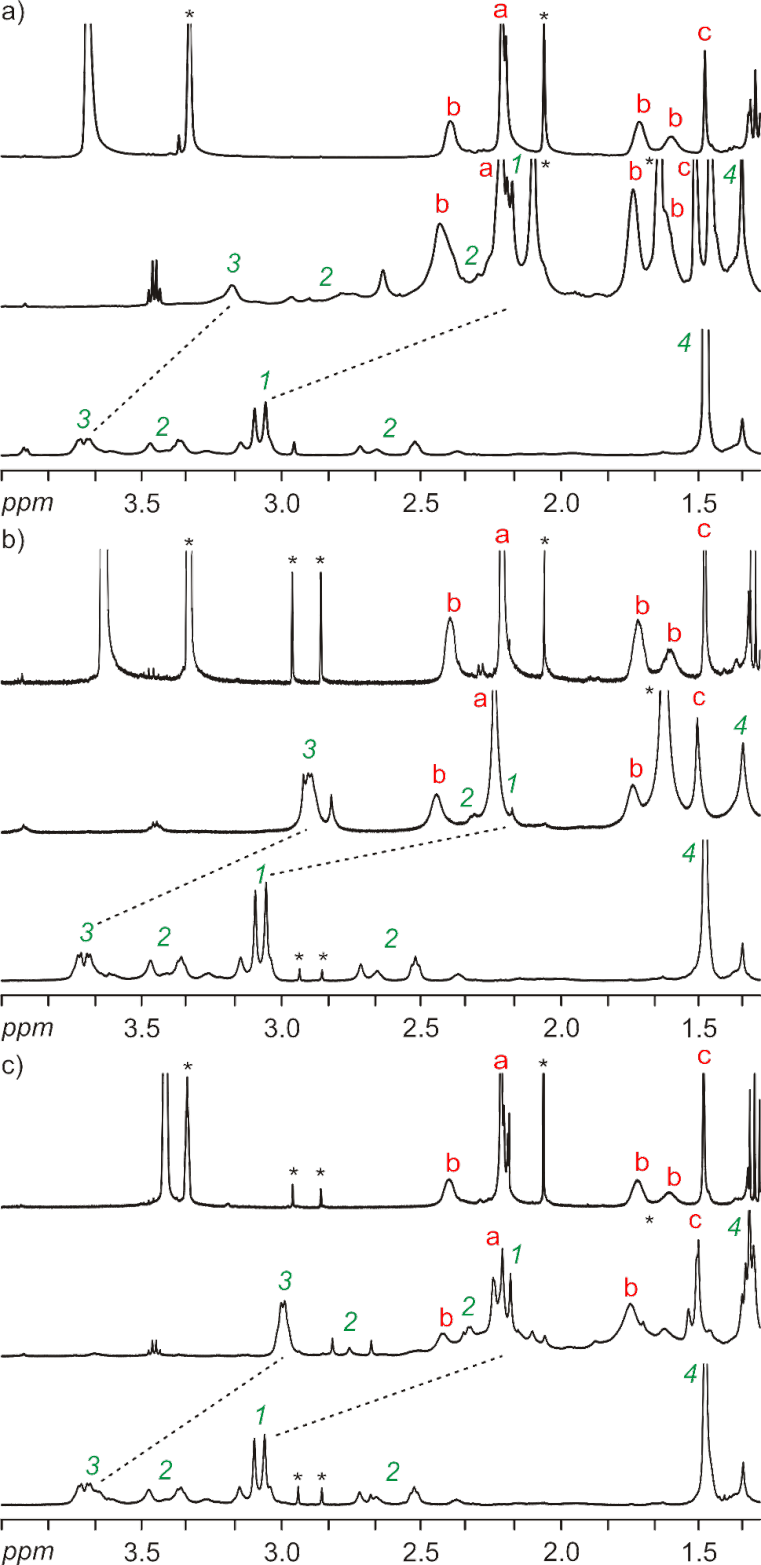
Aliphatic regions of the ^1^H NMR spectra (CD_2_Cl_2_, 500 MHz, 298 K, 2.3 mM) of (a) **10** (top), **17@10** (center) and **17** (bottom), (b) **12** (top), **18@12** (center) and **18** (bottom), and (c) **14** (top), **19@14** (center) and **19** (bottom). Because of the low solubility of the wheel components **10**, **12** and **14**, the samples were dissolved in CD_2_Cl_2_/CD_3_OD (10:1). Red letters assign signals of the wheels as shown in Scheme 2, green numbers those of the axles as shown in Scheme 5.

However, when ^1^H NMR spectra of the free axles are compared with the ^1^H NMR spectra of 1:1 mixtures of axles and wheels, structure-indicative signal shifts are observed that demonstrate the axles to be threaded through the wheels (Figure 2). Consequently, the binding event cannot easily be quantified, but there is qualitative evidence for pseudorotaxane formation. With ^1^H/^1^H-COSY NMR experiments, an assignment of, for example, the *N*-CH_3_ groups to two singlets at ca. 3.1 ppm is possible (protons labelled “1” in the spectra of **17**, **18** and **19** in Figure 2). These signals shift to higher field by ca. 0.9 ppm when 1 equiv of the wheel component is added. These complexation-induced signal shifts are even stronger than similar shifts observed for other rotaxanes with a diamide moiety [101]. The fact that proton “3” also shifts significantly indicates that binding may also involve the carbonyl group of the Boc protective group. A reversible shuttling between both the diamide station and the outer carbonyl group of that moiety and the Boc group would rationalize this shift easily. Consequently, this preliminary NMR evidence qualitatively provides evidence for binding, while a quantification is not easily possible.

## Conclusion

In conclusion, the synthesis of a "toolbox" of multivalent host and guest molecules has been described, which can be obtained from the two easy-to-prepare building blocks **1** and **2** by Sonogashira coupling reactions to ethynyl-substituted spacers. This synthetic approach is convergent, and thus the sometimes limited yields do not detract from this approach. With our toolbox, the number and position of binding sites can be varied systematically; hence, the toolbox provides a means to examine multivalency. However, despite the fact that there is qualitative evidence for pseudorotaxane formation, the binding motif is not yet optimal for a quantitative study. Two problems need to be solved: On one hand, the solubility of the hosts needs to be increased such that a concentration range can be reached that enables us to obtain thermochemical data from ITC experiments. On the other hand, a more suitable binding moiety would be advantageous for use in the axles. The tertiary amides obscure a precise analysis of NMR titrations because of the interconverting *trans* and *cis* amide conformations. Secondary amides again cause solubility problems. However, as described in a recent article [112], diketopiperazines are quite tightly bound to the TLMs. Equipping our wheels with better solubilizing groups and using diketopiperazine axles should therefore help us to go beyond the limitations encountered in the present study.

## Supporting Information

File 1 Experimental details and characterization data.
