# Supplementary material for: Synthesis of multivalent host and guest molecules for the construction of multithreaded diamide pseudorotaxanes
Source: Beilstein J Org Chem. 2012 Feb 9;8:234–45. doi: 10.3762/bjoc.8.24 (PMC3302084; doi:10.3762/bjoc.8.24)
Supplement: File 1 — Experimental details and characterization data. [file Beilstein_J_Org_Chem-08-234-s001.pdf]

Supporting Information

for

Synthesis of multivalent host and guest molecules for the construction of multithreaded diamide pseudorotaxanes

Nora L. Löw[1,#], Egor V. Dzyuba[1,#], Boris Brusilowskij[1,#], Lena Kaufmann[1], Elisa Franzmann[2], Wolfgang Maison[2], Emily Brandt[3,4], Daniel Aicher[3,5], Arno Wiehe[1,3] and Christoph A. Schalley*[,1]

Address: [1]Institut für Chemie und Biochemie der Freien Universität Berlin, Takustr. 3, 14195 Berlin, Germany, [2]Pharmazeutische und Medizinische Chemie, Universität Hamburg, Bundesstr. 45, 20146 Hamburg, Germany, [3]Biolitec research GmbH, Otto-Schott-Str. 15, 07745 Jena, Germany, [4]Present address: Charité – Universitätsmedizin, International Graduate Program Medical Neurosciences, Charitéplatz 1, 10117 Berlin, Germany and [5]Present address: WITEGA Laboratorien Berlin-Adlershof GmbH, Magnusstr. 11, 12489 Berlin, Germany

[#]These three authors contributed equally.

Email: Christoph A. Schalley - christoph@schalley-lab.de

* Corresponding author

Experimental details and characterization data

General

Reagents were purchased from Aldrich, Acros, Lancaster, or Fluka and used without further purification. All reactions were carried out under a protective argon atmosphere. TLM **1**[1], compounds **3**[2], **7**, **14**[1], **15**[3] and **20**[4] were synthesized according to literature procedures. Yields refer to chromatographically and spectroscopically homogeneous

materials. Solvents were dried and distilled prior to use by the usual laboratory methods. Thin-layer chromatography (TLC) was performed on precoated silica gel 60/F254 plates (Merck KGaA). Silica gel (0.04–0.063 mm; Merck) was used for column chromatography.

NMR spectroscopy: ^{1}H and ^{13}C spectra were obtained on Bruker AC 250 (^{1}H: 250 MHz; ^{13}C: 62.5 MHz), Bruker ECX 400 (^{1}H: 400 MHz; ^{13}C: 101 MHz), Jeol ECP 500 (^{1}H: 500 MHz; ^{13}C: 125 MHz) or Bruker AVANCE III 700 (^{1}H: 700 MHz; ^{13}C: 220 MHz) instruments at 298 K. All chemical shifts are reported in ppm with the signals of residual solvent protons taken as internal standards; coupling constants are in Hz. The following abbreviations were used to indicate NMR-multiplicities: s (singlet), d (doublet), t (triplet), q (quartet), m (multiplet), br (broad). Binding experiments were carried out in CD_2Cl_2 at 298 K on a Bruker ECX 400 or on a Jeol ECP 500 instrument.

Mass spectrometry: ESI-TOF mass spectra were obtained on an Agilent 6210 ESI-TOF, Agilent Technologies, Santa Clara, CA, USA. The solvent flow rate was adjusted to 4 μL/min and the spray voltage set to 4 kV. The drying gas flow rate was adjusted to 15 psi (1 bar). All other parameters were optimized for maximum abundance of the $[M + H]^+$, $[M + Na]^+$ or $[M + K]^+$ ions. ESI-FT-ICR mass spectra were obtained on an Ionspec QFT-7, Varian Inc., Lake Forest, CA, USA, equipped with a 7 T magnet and a micromass Z-spray ESI source, Waters Co., Saint-Quentin, France. The solvent flow rate was adjusted to 4 μL/min and the spray voltage set to 3.8 kV. All other parameters were optimized for maximum abundance of the corresponding quasimolecular ions.

Benzyl {2-[(3-*tert*-butoxycarbonylamino)propanamido]ethyl} *N*-methylcarbamate (5): Benzyl methyl[2-(methylamino)ethyl]carbamate (**3**) (231 mg, 1.04 mmol) and *N*-(*tert*-butoxycarbonyl)-ß-alanine (**4**) (255 mg, 1.35 mmol) were dissolved in DMF (6 mL). After cooling to 0 °C, the reaction mixture was treated with EDC (0.24 mL, 1.35 mmol) and HOBt (70 mg, 0.52 mmol), warmed up to rt and stirred for 22 h. The solvent was evaporated under reduced pressure and the oily residue taken up in EtOAc. The organic layer was washed with saturated sodium bicarbonate solution (2×), and brine (2×), dried over $MgSO_4$ and evaporated to dryness. The product **5** was formed as a yellow oil and was used without further purification (488 mg, 92%). ^{1}H NMR (250 MHz, $CDCl_3$) δ 1.39 (s, 9H; $(CH_3)_3C$), 2.37 (br, 2H; CH_2), 2.93 (br, 3H; CH_3), 2.94 (s, 3H; CH_3), 3.42 (br, 6H; CH_2), 5.10 (s, 2H; CH_2), 7.32–7.34 ppm (m, 5H; ArH); ^{13}C NMR (62.5 MHz, $CDCl_3$) δ 28.4, 32.7, 32.8, 33.6, 34.5, 35.1, 35.4, 36.4, 37.0, 45.1, 45.9, 47.3, 47.7, 66.9, 79.7, 127.8, 127.9, 128.6, 136.8, 156.0, 156.5,

172.0 ppm; ESIMS *m*/*z* (%): 394.2 (100) $[M + H]^+$; HRMS (FT-ICR-ESI$^+$) *m*/*z*: $[M + H]^+$ calcd for $C_{20}H_{31}N_3O_5^+$, 394.2336; found, 394.2354.

***tert*-Butyl 3-(methyl(2-(methylamino)ethyl)amino)-3-oxopropylcarbamate (6):** Pd/C (120 mg, 10 wt %) was placed in EtOH (25 mL) and treated with the Cbz-protected amine **5** (350 mg, 0.89 mmol). The reaction mixture was then hydrogenated for 3 d under normal pressure and after that filtered over celite to remove the catalyst. The solution was evaporated under reduced pressure, and the product **6** (226 mg, 98%) was obtained as a yellow oil without further purification. ^{1}H NMR (250 MHz, $(CD_3)_2CO$) δ 1.39 (s, 9H; CH_3), 2.50–2.56 (m, 2H; CH_2), 2.90 (m, 6H; CH_3), 3.25–3.47 (m, 6H; CH_2), 5.98 ppm (br, 1H; NH); ^{13}C NMR (62.5 MHz, $(CD_3)_2CO$) δ 27.9, 32.7, 32.8, 33.4, 35.2, 35.4, 35.8, 36.2, 36.3, 46.8, 48.9, 49.1, 49.6, 77.9, 155.9, 171.7 ppm; ESIMS *m*/*z* (%): 260.2 (100) $[M + H]^+$; HRMS (FT-ICR-ESI$^+$) *m*/*z*: $[M + H]^+$ calcd for $C_{12}H_{25}N_3O_3^+$, 260.1968; found, 260.1970.

4'-Iodobiphenyl-3-carboxylic acid (8): Biphenyl-3-carboxylic acid (**7**) (300 mg, 1.50 mmol), I_2 (220 mg, 0.90 mmol) and PIDA (289 mg, 0.90 mmol) were dissolved in a mixture of AcOH and Ac_2O (3 mL, 1:1 v/v) and stirred at rt for 1 h. Saturated sodium thiosulfate solution (2 mL) and $(NH_4)_2CO_3$ (700 mg) were then added and the reaction mixture was stirred for a further 10 min. The resulting solid was filtered off, washed with water and dried vigorously in vacuo. The product **8** (328 mg, 67%) was obtained as a grey solid without further purification. ^{1}H NMR (400 MHz, $(CD_3)_2SO$) δ 7.50–7.53 (m, 3H; ArH), 7.62 (t, ^3J = 7.5 Hz, 1H; ArH), 7.83–7.85 (m, 2H; ArH), 7.90–7.96 (m, 1H; ArH), 8.16 (br, 1H; ArH) 13.12 ppm (br, 1H; C(=O)OH); ^{13}C NMR (101 MHz, $(CD_3)_2SO$) δ 94.4, 127.1, 128.6, 129.4, 131.6, 137.8, 138.7, 139.4, 167.1 ppm; ESIMS *m*/*z* (%): 322.9 (100) $[M-H]^-$; HRMS (FT-ICR-ESI$^-$) *m*/*z*: $[M-H]^-$ calcd for $C_{13}H_9IO_3^-$, 322.9569; found, 322.9590.

***tert*-Butyl 3-((2-(4'-iodo-*N*-methylbiphenyl-3-ylcarboxamido)ethyl)(methyl)amino)-3-oxopropylcarbamate (diamide axle piece, 2):** Amine **6** (153 mg, 0.59 mmol) and acid **8** (150 mg, 0.46 mmol) were dissolved in DMF (5 mL) and the solution was cooled down to 0 °C. EDC·HCl (115 mg, 0.60 mmol) and HOBt (31 mg, 0.23 mmol) were then added, and the reaction mixture was warmed up to rt and stirred for 24 h. The solvent was evaporated under reduced pressure and the residue taken up in EtOAc. The organic layer was washed with saturated sodium bicarbonate solution (2×), and brine (2×), dried over $MgSO_4$ and evaporated to dryness. The product **2** was obtained after column chromatography (silica gel, CH_2Cl_2/MeOH 49:1 v/v) from the first fraction as a slightly yellow oil (190 mg, 73%).

^{1}H NMR (400 MHz, CD_2Cl_2) δ 1.39 (s, 9H; $(CH_3)_3C$), 2.46–2.50 (m, 2H; CH_2), 2.65–2.71 (m, 2H; CH_2), 3.00 (s, 3H; CH_3), 3.05 (s, 3H; CH_3), 3.40–3.43 (m, 2H; CH_2), 3.60–3.70 (m, 2H; CH_2), 5.46 (br, 1H; NH), 7.33–7.38 (m, 3H; ArH), 7.45–7.51 (m, 1H; ArH), 7.58–7.63 (m, 2H; ArH), 7.77–7.81 ppm (m, 2H; ArH); ^{13}C NMR (101 MHz, $CDCl_3$) δ 28.5, 33.8, 35.8, 36.4, 38.2, 44.6, 45.0, 79.0, 125.4, 126.1, 128.0, 128.9, 137.1, 138.0, 139.8, 140.3, 156.0, 171.3 ppm; ESIMS *m/z* (%): 588.1 (100) $[M + Na]^+$; HRMS (FT-ICR-ESI$^+$) *m/z*: $[M + Na]^+$ calcd for $C_{25}H_{32}IN_3O_3Na^+$, 588.1329; found, 588.1336.

Divalent host 10: TLM **1** (200.0 mg, 0.184 mmol) and 1,3-diethynyl benzene (**9**) (11.3 mg, 0.09 mmol) were dissolved in a mixture of DMF (10 mL) and NEt_3 (3 mL). The solution was degassed for 1 h and protected from light. Afterwards, PPh_3 (5.4 mg, 18 μmol), $(Ph_3P)_2PdCl_2$ (6.3 mg, 9 μmol) and CuI (1.8 mg, 9 μmol) were added. The resulting mixture was stirred at rt for 24 h. The solvents were evaporated under reduced pressure, and the desired product **10** was obtained after chromatographic work-up (silica gel, CH_2Cl_2/EtOAc 12:1 to 4:1 v/v) from the third fraction as a slightly brown solid (159.0 mg, 90%). ^{1}H NMR (500 MHz, $CDCl_3/CD_3OD$ 10:1 v/v) δ 1.38 (s, 18H; $(CH_3)_3C$), 1.50 (br, 8H; CH_2), 1.62 (br, 16H; CH_2), 2.12 (br, 48H; $ArCH_3$), 2.32 (br, 16H; CH_2), 6.99 (2 s, 16H; ArH), 7.38 (br, 2H; ArH), 7.54 (br, 1H; ArH), 7.73 (s, 1H; ArH), 8.13 (s, 4H; ArH), 8.17 (s, 6H; ArH), 8.23 ppm (br, 2H; ArH); ^{13}C NMR (125 MHz, $CDCl_3/CD_3OD$ 10:1 v/v) δ 18.3, 22.7, 25.0, 26.1, 29.4, 30.8, 44.9, 88.1, 90.1, 121.9, 123.8, 124.6, 125.9, 126.0, 128.2, 128.6, 131.0, 131.1, 133.6, 133.9, 134.7, 134.8, 147.7, 147.9, 153.1, 165.6, 166.6 ppm; ESIMS *m/z* (%): 2146.3 (100) $[M + HNEt_3]^+$; HRMS (FT-ICR-ESI$^+$) *m/z*: $[M + HNEt_3]^+$ calcd for $C_{144}H_{162}N_9O_8^+$, 2146.2579; found, 2146.2570. Electrospray ionization of the macrocycles is most easily accomplished in the form of the corresponding $HNEt_3^+$ complexes when a small amount of triethylamine is added to the sample solution. See also reference [1].

Divalent host 12: TLM **1** (200.0 mg, 0.184 mmol) and 1,4-diethynyl benzene (**11**) (11.3 mg, 0.09 mmol) were dissolved in a mixture of DMF (10 mL) and NEt_3 (3 mL). The solution was degassed for 1 h and protected from light. Afterwards, PPh_3 (5.4 mg, 18 μmol), $(Ph_3P)_2PdCl_2$ (6.3 mg, 9 μmol) and CuI (1.8 mg, 9 μmol) were added. The resulting mixture was stirred at rt for 24 h. The solvents were evaporated under reduced pressure and the desired product **12** was obtained after chromatographic work-up (silica gel, CH_2Cl_2/EtOAc 12:1 to 6:1 v/v) from the third fraction as a yellow solid (129.0 mg, 78%). ^{1}H NMR (500 MHz, $CDCl_3/CD_3OD$ 10:1 v/v) δ 1.38 (s, 18H; $(CH_3)_3C$), 1.52 (br, 8H; CH_2), 1.63 (br, 16H; CH_2), 2.13 (br s, 48H;

$ArCH_3$), 2.23 (br, 16H; CH_2), 6.99 (2 s, 16H; ArH), 7.53 (br, 4H; ArH), 8.10 (s, 2H; ArH), 8.14 (s, 4H; ArH), 8.24 (s, 4H; ArH), 8.26 ppm (s, 2H; ArH); ^{13}C NMR (125 MHz, $CDCl_3/CD_3OD$ 10:1 v/v) δ 18.1, 22.7, 26.4, 24.8, 29.3, 30.9, 44.9, 87.7, 92.4, 122.0, 122.4, 123.7, 124.2, 124.9, 125.1, 126.3, 128.6, 128.9 131.0, 131.3, 133.7, 134.1, 134.5, 134.8, 147.4, 148.0, 153.3, 165.4, 165.9 ppm; ESIMS *m*/*z* (%): 2146.3 (100) $[M + HNEt_3]^+$; HRMS (FT-ICR-ESI$^+$) *m*/*z*: $[M + HNEt_3]^+$ calcd for $C_{144}H_{162}N_9O_8^+$, 2146.2579; found, 2146.2581.

Tetravalent host 16: TLM **1** (75.3 mg, 0.184 mmol) and Zn porphyrin **15** (5.4 mg, 0.011 mmol) were dissolved in a mixture of DMF (15 mL) and NEt_3 (5 mL). The solution was degassed for 1 h and protected from light. Afterwards, $AsPh_3$ (15.2 mg, 50 μmol) and $Pd_2(dba)_3$ (5.3 mg, 6 μmol) were added. The resulting mixture was stirred at 120 °C for 12 h in a sealed tube. The solvents were evaporated under reduced pressure and the desired product **16** was obtained after chromatographic work-up (silica gel, CH_2Cl_2/MeOH 24:1 v/v) from the green-coloured fraction as a green solid (2.0 mg, 7%). ^1H NMR (400 MHz, $CDCl_3/CD_3OD$ 9:1 v/v) δ 1.33 (s, 36H; $(CH_3)_3C$), 1.45 (br, 16H; CH_2), 1.57 (br, 32H; CH_2), 2.28 (br s, 48H; $ArCH_3$), 2.27 (br, 32H; CH_2), 6.93 (br, 32H; ArH), 8.12 (s, 8H; ArH), 8.36 (s, 4H; ArH), 8.70 (s, 8H; ArH), 8.78 (s, 4H; ArH), 9.62 ppm (br, 8H; ArH); ESIMS *m*/*z* (%): 2153.7 (100) $[M + 2H]^{2+}$.

Divalent axle 17: Compound **2** (150 mg, 0.270 mmol) and 1,3-diethynyl benzene (**9**) (15.3 mg, 0.12 mmol) were dissolved in a mixture of DMF (3 mL) and NEt_3 (2 mL). The solution was degassed for 1 h and protected from light. Afterwards, PPh_3 (15.6 mg, 52 μmol), $Pd_2(dba)_3$ (23.8 mg, 26 μmol) and CuI (5.2 mg, 26 μmol) were added. The resulting mixture was stirred at 70 °C for 3 d. The solvents were evaporated under reduced pressure and the desired product **17** was obtained after chromatographic work-up (silica gel, CH_2Cl_2/MeOH 30:1 v/v) from the third fraction as a brown solid (39.0 mg, 32%). ^1H NMR (400 MHz, CD_2Cl_2) δ 1.38 (s, 18H; $(CH_3)_3C$), 2.44–2.66 (m, 4H; CH_2), 3.01, 3.05, 3.10 (3 s, 12H; CH_3), 3.30–3.46 (m, 4H; CH_2), 3.65–3.70 (m, 8H; CH_2), 5.35 (br, 2H; NH), 7.34–7.40 (m, 4H; ArH), 7.47–7.54 (m, 4H; ArH), 7.63–7.70 ppm (m, 12H; ArH); ^{13}C NMR (125 MHz, CD_2Cl_2) δ 28.2, 29.8, 33.0, 33.7, 35.6, 36.5, 38.0, 44.6, 49.7, 78.7, 89.4, 89.8, 122.4, 122.5, 123.7, 125.4, 126.1, 127.1, 127.2, 127.9, 128.8, 129.0, 131.5, 132.2, 134.5, 137.5, 140.4, 155.8, 171.1, 172.4 ppm; ESIMS *m*/*z* (%): 1023.5 (100) $[M + Na]^+$; HRMS (TOF-ESI$^+$) *m*/*z*: $[M + Na]^+$ calcd for $C_{60}H_{68}N_6O_8Na^+$, 1023.4991; found, 1023.4958.

Divalent axle 18: Compound **2** (150 mg, 0.270 mmol) and 1,4-diethynyl benzene (**11**) (15.3 mg, 0.12 mmol) were dissolved in DMF (3 mL) and NEt_3 (2 mL). The solution was degassed for 1 h and protected from light. Afterwards, PPh_3 (15.6 mg, 52 μmol), $Pd_2(dba)_3$ (23.8 mg, 26 μmol) and CuI (5.2 mg, 26 μmol) were added. The resulting mixture was stirred at 70 °C for 3 d. The solvents were evaporated under reduced pressure and the desired product **18** was obtained after chromatographic work-up (silica gel, CH_2Cl_2/MeOH 50:1 v/v) from the third fraction as a slightly yellow solid (44.3 mg, 37%). ^{1}H NMR (400 MHz, CD_2Cl_2) δ 1.38 (s, 18H; $(CH_3)_3C$), 2.44–2.65 (m, 4H; CH_2), 3.00, 3.04, 3.10 (3 s, 12H; CH_3), 3.32–3.43 (m, 4H; CH_2), 3.65–3.70 (m, 8H; CH_2), 5.35 (br, 2H; NH), 7.34 (d, ^3J = 7.7 Hz, 2H; ArH), 7.48 (t, ^3J = 14.5 Hz, 2H; ArH), 7.54 (s, 4H; ArH), 7.63–7.69 ppm (m, 12H; ArH); ^{13}C NMR (125 MHz, $CDCl_3/CD_3OD$ 15:1 v/v) δ 28.1 29.5, 33.5, 35.4, 36.0, 37.9, 44.5, 44.9, 49.8, 79.1, 90.0, 90.9, 122.3, 123.0, 125.2, 125.7, 126.8, 126.9, 128.0, 128.9, 131.4, 132.0, 136.4, 139.9, 140.4, 156.1, 171.6, 172.6 ppm; ESIMS *m*/*z* (%): 1023.5 (100) $[M + Na]^+$; HRMS (TOF-ESI$^+$) *m*/*z*: $[M + Na]^+$ calcd for $C_{60}H_{68}N_6O_8Na^+$, 1023.4991; found, 1023.4960.

Trivalent axle 19: Compound **2** (150 mg, 0.270 mmol) and 1,3,5-triethynyl benzene (**11**) (12.3 mg, 0.8 mmol) were dissolved in a mixture of DMF (3 mL) and NEt_3 (2 mL). The solution was degassed for 1 h and protected from light. Afterwards, PPh_3 (15.6 mg, 52 μmol), $Pd_2(dba)_3$ (23.8 mg, 26 μmol) and CuI (5.2 mg, 26 μmol) were added. The resulting mixture was stirred at 70 °C for 3 d. The solvents were evaporated under reduced pressure and the desired product **19** was obtained after chromatographic work-up (silica gel, CH_2Cl_2/MeOH 18:1 v/v) from the third fraction as a slightly yellow solid (29.0 mg, 24%). ^{1}H NMR (400 MHz, CD_2Cl_2) δ 1.38 (s, 27H; $(CH_3)_3C$), 2.44–2.66 (m, 6H; CH_2), 3.01, 3.04, 3.10 (3 s, 18H; CH_3), 3.32–3.43 (m, 6H; CH_2), 3.62–3.70 (m, 12H; CH_2), 5.35 (br, 3H; NH), 7.31–7.37 (m, 4H; ArH), 7.47–7.53 (m, 3H; ArH), 7.63–7.71 ppm (m, 21H; ArH); ^{13}C NMR (125 MHz, CD_2Cl_2) δ 27.5, 29.1, 33.0, 34.9, 35.8, 37.3, 44.0, 44.3, 48.0, 78.0, 87.9, 89.8, 121.4, 121.5, 123.5, 124.7, 125.4, 126.5, 127.2, 128.4, 131.6, 133.4, 136.3, 136.8, 139.7, 139.9, 155.2, 170.5, 171.7 ppm; ESIMS *m*/*z* (%): 1485.7 (100) $[M + Na]^+$; HRMS (TOF-ESI$^+$) *m*/*z*: $[M + Na]^+$ calcd for $C_{87}H_{99}N_9O_{12}Na^+$, 1485.7338; found, 1485.7329.

Trivalent axle 21: Compound **2** (87.0 mg, 0.150 mmol) and triethynyl adamantane (**20**) (9.7 mg, 0.05 mmol) were dissolved in a mixture of DMF (8 mL) and NEt_3 (3 mL). The solution was degassed for 1 h and protected from light. Afterwards, PPh_3 (8.7 mg, 29 μmol), $Pd_2(dba)_3$ (13.2 mg, 14 μmol) and CuI (2.9 mg, 14 μmol) were added. The resulting mixture

was stirred at 70 °C for 3 d. The solvents were evaporated under reduced pressure and the desired product **21** was obtained after chromatographic work-up (silica gel, eluting first with EtOAc, then EtOAc/MeOH 25:3 to CH_2Cl_2/MeOH 10:1 v/v) from the third fraction as a slightly brown oil (45.0 mg, 64%). ^{1}H NMR (400 MHz, $CDCl_3/CD_3OD$ 9:1 v/v) δ 1.27 (s, 27H; $(CH_3)_3C$), 1.83 (br, 4H; CH_2), 2.05 (br, 4H; CH_2), 2.14 (s, 1H; CH), 2.38–2.41 (m, 6H; CH_2), 2.52–2.58 (m, 4H; CH_2), 2.95, 2.97 (2 s, 18H; CH_3), 3.19–3.22 (m, 8H; CH_2), 3.45–3.50 (m, 4H; CH_2), 7.19 (br, 4H; ArH), 7.31–7.37 (m, 8H; ArH), 7.38–7.43 (m, 6H; ArH), 7.47 (s, 2H; ArH), 7.52 ppm (br, 4H; ArH); ^{13}C NMR (125 MHz, $CDCl_3/CD_3OD$ 9:1 v/v) δ 14.1, 27.3, 28.0, 33.3, 35.5, 38.0, 40.4, 44.8, 46.3, 79.1, 96.3, 122.8, 125.0, 126.5, 128.0, 128.7, 131.9, 135.9, 138.9, 140.4, 162.9, 171.8, 179.1 ppm; ESIMS *m*/*z* (%): 1543.8 (100) $[M + Na]^+$; HRMS (FT-ICR-ESI$^+$) *m*/*z*: $[M + Na]^+$ calcd for $C_{91}H_{109}N_9O_{12}Na^+$, 1543.8120; found, 1543.8146.

Tetravalent axle 23: Compound **2** (90.0 mg, 0.160 mmol) and Zn porphyrin **15** (5.4 mg, 0.011 mmol) were dissolved in DMF (5 mL) and NEt_3 (5 mL). The solution was degassed for 1 h and protected from light. Afterwards, $AsPh_3$ (34.9 mg, 0.11 mmol) and $Pd_2(dba)_3$ (12.1 mg, 13 μmol) were added. The resulting mixture was stirred at 80 °C for 2 d. The solvents were evaporated under reduced pressure and the desired product **23** was obtained after chromatographic work-up (silica gel, CH_2Cl_2/MeOH 23:2 v/v) from the fourth fraction as a green solid (10.0 mg, 17%). ^{1}H NMR (400 MHz, CD_2Cl_2/CD_3OD 9:1 v/v) δ 1.33 (s, 36H; $(CH_3)_3C$), 2.46–2.53 (m, 8H; CH_2), 2.60–2.70 (m, 6H; CH_2), 3.04, 3.08 (2 s, 18H; CH_3), 3.31–3.36 (m, 8H; CH_2), 3.60–3.70 (m, 8H; CH_2), 7.31–7.34 (m, 4H; ArH), 7.49–7.78 (m, 24H; ArH), 8.01 (d, ^3J = 7.5 Hz, 7H; ArH), 9.33 ppm (br, 8H; ArH); ^{13}C NMR (125 MHz, CD_2Cl_2/CD_3OD 9:1 v/v) δ 28.2, 30.3, 32.6, 34.3, 36.2, 37.1, 38.7, 79.0, 94.4, 97.1, 124.6, 126.0, 127.9, 129.0, 130.4, 131.3, 132.0, 132.9, 137.6, 141.4, 151.6, 157.2, 172.6 ppm; ESIMS *m*/*z* (%): 2242.0 (100) $[M + Na]^+$.

Divalent axle 22: This compound was obtained as a side product from the synthesis of tetravalent axle **23** after chromatographic work-up (silica gel, CH_2Cl_2/MeOH 23:2 v/v) from the third fraction as a slightly brownish oil (8.6 mg, 37%). ^{1}H NMR (400 MHz, CD_2Cl_2) δ 1.37 (s, 18H; $(CH_3)_3C$), 2.45–2.50 (m, 4H; CH_2), 3.00 (s, 6H; CH_3), 3.32 (s, 6H; CH_3), 3.30–3.40 (m, 6H; CH_2), 3.60–3.70 (m, 6H; CH_2), 7.31–7.34 (m, 1H; ArH), 7.49–7.51 (m, 2H; ArH), 7.65 (br, 1H; ArH), 7.70–7.77 ppm (m, 12H; ArH); ^{13}C NMR (101 MHz, CD_2Cl_2) δ 28.2, 33.7, 35.5, 36.5, 38.0, 44.6, 44.9, 79.0, 125.3, 125.7, 127.5, 127.8, 137.4, 139.4, 139.7,

155.8, 172.3 ppm; ESIMS *m*/*z* (%): 877.5 (100) $[M + H]^+$; HRMS (FT-ICR-ESI$^+$) *m*/*z*: $[M + H]^+$ calcd for $C_{50}H_{65}N_6O_8^+$, 877.4858; found, 877.4921.

References

1) Baytekin, B.; Zhu, S. S.; Brusilowskij, B.; Illigen, J.; Ranta, J.; Huuskonen, J.; Russo, L.; Rissanen, K.; Kaufmann, L.; Schalley, C. A. *Chem. Eur. J.* **2008**, *14*, 10012-10028.

2) H.-X. Liu, F. Shao, G.-Q. Li, G.-L. Xun, Z.-J. Yao, *Chem. Eur. J.* **2008**, *14*, 8632-8639.

3) Kabalka, G. W.; Wang, L. *Tetrahedron Lett.* **2002**, *43*, 3067-3068.

4) Ljungdahl, T.; Bennur, T.; Dallas, A.; Emtenäs, H.; Mårtensson, J. *Organometallics* **2008**, *27*, 2490-2498.
